# Frontline assessors’ opinions about grading committees in a medicine clerkship

**DOI:** 10.1186/s12909-024-05604-x

**Published:** 2024-06-05

**Authors:** Sophia K. Lewis, Nathanial S. Nolan, Lisa Zickuhr

**Affiliations:** 1 Department of Medicine, Washington University School of Medicine, St. Louis, MO USA; 2 Division of Infectious Disease, VA St Louis Health Care System, St. Louis, MO USA; 3 Division of Infectious Disease, Department of Medicine, Washington University School of Medicine, St. Louis, MO USA; 4 Division of Rheumatology, Department of Medicine, Washington University School of Medicine, St. Louis, USA

**Keywords:** Grading committee, Clinical assessment, Medical education, Internal medicine clerkship

## Abstract

**Background:**

Collective decision-making by grading committees has been proposed as a strategy to improve the fairness and consistency of grading and summative assessment compared to individual evaluations. In the 2020–2021 academic year, Washington University School of Medicine in St. Louis (WUSM) instituted grading committees in the assessment of third-year medical students on core clerkships, including the Internal Medicine clerkship. We explored how frontline assessors perceive the role of grading committees in the Internal Medicine core clerkship at WUSM and sought to identify challenges that could be addressed in assessor development initiatives.

**Methods:**

We conducted four semi-structured focus group interviews with resident (*n* = 6) and faculty (*n* = 17) volunteers from inpatient and outpatient Internal Medicine clerkship rotations. Transcripts were analyzed using thematic analysis.

**Results:**

Participants felt that the transition to a grading committee had benefits and drawbacks for both assessors and students. Grading committees were thought to improve grading fairness and reduce pressure on assessors. However, some participants perceived a loss of responsibility in students’ grading. Furthermore, assessors recognized persistent challenges in communicating students’ performance via assessment forms and misunderstandings about the new grading process. Interviewees identified a need for more training in formal assessment; however, there was no universally preferred training modality.

**Conclusions:**

Frontline assessors view the switch from individual graders to a grading committee as beneficial due to a perceived reduction of bias and improvement in grading fairness; however, they report ongoing challenges in the utilization of assessment tools and incomplete understanding of the grading and assessment process.

**Supplementary Information:**

The online version contains supplementary material available at 10.1186/s12909-024-05604-x.

## Background

Undergraduate medical education programs utilize summative assessments to compare student performance against defined learning objectives, judging whether students have achieved the knowledge, attitudes, and skills needed to successfully complete their current course or clerkship and transition to the next phase of their training [1]. This system upholds the importance of patients as stakeholders in medical education, and it ensures trainees can competently deliver care to the extent that their phase of training permits [1]. Medicine clerkship grades, a form of summative assessment, serve as indicators of student achievement relative to the clerkship’s pre-determined competencies and provide feedback to students on their clinical skills and knowledge [2–4]. At many medical schools, Internal Medicine clerkship grades are based on a combination of standardized written tests, objective structured clinical examinations, and workplace performance assessments, which are completed by faculty and residents with particular emphasis on direct observations of clinical performance [5].

Ideally summative assessment systems, including clerkship grades, would ensure that medical schools graduate competent physicians ready for the next phase of training in a manner free of bias. However, there is growing concern about grading accuracy and fairness from both students and clinical supervisors, especially in light of the high value placed on these grades during awards and residency program selection processes [2, 6]. The challenge of grading reliability stems, in part, from inter-institutional and inter-clerkship variability in grading practices, as well as interrater differences in subjective judgement of student performance [7–10]. Furthermore, increasing evidence suggests gender and racial bias contribute to grading discrepancies, including at our own institution, Washington University School of Medicine in St. Louis (WUSM) [11–17].

Collective decision-making by grading committees has been proposed as a strategy to improve the fairness, transparency and consistency of grading compared to individual grader assessment [6]. Moreover, implementation of grading committees allows for a holistic discussion of student performance, with internal support for difficult decisions [18]. In essence, shared decisions are thought to be superior to decisions made by individuals [19]. This strategy has already been adopted in graduate medical education (GME), with assessment of resident and fellow physician performance occurring via Clinical Competency Committees [19].

In 2020–2021, WUSM instituted grading committees in the assessment of medical students on core clerkships. Before then, clerkship workplace performance assessments consisted of written and verbal evaluations. Supervising faculty and residents were also asked to submit a final grade to the Clerkship Director regarding the student’s clinical performance, based on a grading system of honors, high pass, pass, or fail. The Clerkship Director would then finalize clinical grades based on the composite of assessment data. The WUSM Internal Medicine grading committee introduced in 2020–2021 was composed of eight clinician educators representing a diversity of backgrounds and multiple specialties, such as Primary Care, Community and Public Health, Hospital Medicine, Infectious Diseases, and Rheumatology. All members had existing expertise in medical education and assessment, and all members underwent unconscious bias training.

With the introduction of grading committees, frontline assessors, defined as the faculty and residents who supervise medical students in clinical settings, submit assessment data via standardized forms every two weeks. The assessment form utilized in the 2020–2021 academic year started with two general comment boxes asking for global narrative feedback on what the student did well and where they could improve. Next, there was a series of 14 prompts about key domains including medical knowledge, patient care, interpersonal and communication skills, professionalism, and practice-based learning and improvement. Each prompt asked assessors to select descriptors from a list of 4–14 options that best matched the behaviors they observed over the two-week rotation. Grading committees synthesize de-identified assessment data from multiple assessors to assign final clerkship grades.

Of note, WUSM underwent curriculum reform and welcomed the Gateway Internal Medicine clerkship in January 2022. The Gateway Internal Medicine clerkship introduced a new competency-based assessment system that continues to employ grading committees but differs in how assessment data are collected and the grades students may earn [20]. Within this article, we focus on the former curriculum and specify when lessons learned were applied to the Gateway clerkship.

While the effect of group decision-making on grading fairness is being explored, less is known about the impact of this change on the roles of frontline assessors. In this study, we investigate the use of grading committees in summative assessment decisions, aiming to (1) explore frontline assessors’ opinions about the benefits and challenges of the new grading committee process at WUSM and to (2) understand faculty and resident comfort performing the workplace-based assessments utilized by grading committees to best inform faculty development initiatives at our institution.

## Methods

### Design

We conducted a qualitative methods study with conventional thematic analysis [21, 22]. We utilized semi-structured focus group interviews to explore the views of our participants. The study was approved by the Institutional Review Board at WUSM (IRB #202,102,048).

### Setting

We conducted this study among assessors involved in the Internal Medicine core clerkship at WUSM and affiliated teaching hospitals, Barnes-Jewish Hospital (BJH) and John Cochran Veterans Affairs Medical Center (VAMC) in St. Louis, Missouri. Focus groups were held from February to April 2021, at the conclusion of the first academic year using grading committees. Focus groups were conducted virtually on WUSM’s HIPAA compliant Zoom platform.

### Sampling and participants

We invited frontline assessors, supervising residents and faculty from both inpatient and outpatient educational sites within the Internal Medicine clerkship, to participate in semi-structured focus groups. Invited attending physicians were educators who supervise medical students in clinical settings. Invited residents were in their PGY-2 or PGY-3 years, as upper-level residents participate in medical student assessment on clerkship rotations. To best bring the general opinions of assessors to the surface for informing faculty development initiatives, grading committee members were excluded from volunteering as interviewees. Grading committee members, who are intimately knowledgeable about the grading committee process, participated as focus group moderators to facilitate honest discussion in the absence of clerkship leadership.

Standardized IRB-approved emails inviting participants to volunteer were sent to existing listservs of teaching faculty and residents (convenience sampling). A total of four focus groups were conducted with four separate participant clusters: resident physicians, attending physicians from a variety of outpatient disciplines, and attending physicians from inpatient rotations at BJH and VAMC. Participants volunteered in response to recruitment emails. An IRB-approved consent document was emailed to all potential volunteers, and informed verbal consent was obtained at the start of each focus group meeting.

### Data collection

Focus group questions were designed by research team members (LZ, SL) to investigate multiple facets of grading committees and identify pitfalls most amenable to faculty and resident development at our institution. Questions were fine-tuned through a collaborative, deductive approach among medical education leadership, including the Assistant Dean of Assessment and Associate Dean for Medical Student Education. Final interview questions were revised based on feedback from a mock interview with focus group leaders. Questions covered assessors’ understanding of the grading committee process, perceived and ideal assessor roles, and the benefits and drawbacks of the grading committee (see Additional File 1). Facilitators were permitted to ask probing follow-up questions to clarify and expand on comments. We continued to host focus groups until assessors from each of the major teaching services had the opportunity to participate and our data set reached saturation with no new themes identified [23].

Interviews were moderated by one lead discussant with a secondary moderator present to ask additional clarifying questions. Moderators consisted of one junior resident (SL) and three grading committee faculty members (JC, CM, IR). Focus group discussions were recorded and professionally transcribed (www.rev.com/). Transcripts were de-identified prior to qualitative analysis.

### Data analysis

Qualitative data analysis was organized using the commercial online software Dedoose (Dedoose Version 9.0.17, web application for managing, analyzing, and presenting qualitative and mixed method research data, 2021. Los Angeles, CA: SocioCultural Research Consultants, LLC, www.dedoose.com). Transcripts were independently reviewed by two researchers (SL, NN) to generate an initial code book based on identified commonalities and patterns within focus group responses. The code book was refined by an iterative process of discussion and transcript review. Both researchers independently applied the final code book to all four transcripts. Coding differences were subsequently resolved through group discussion with a third researcher (LZ) until consensus was achieved. Final coded excerpts were reviewed by all three researchers (LZ, SL, NN), which included an attending representative from clerkship leadership, a resident, and a frontline assessor. All coders had advanced training in medical education and represented different roles within medical education, providing a diversity of perspectives. Connections between codes were linked into overarching and interconnecting themes. All authors agreed upon the final codes and themes.

## Results

### Participant characteristics

Of an estimated 230 assessors, twenty-three volunteers participated in our study across four focus groups (Table 1). At the resident physician level, both PGY-2 and PGY-3 residents were represented, as PGY-1 residents do not assess WUSM students. At the faculty level, participants ranged in seniority from Instructor to Professor. Faculty represented Internal Medicine subspecialities, Primary Care, and Hospitalist Medicine. Participants’ primary teaching environments included inpatient Medicine, inpatient Cardiology, and outpatient Primary Care or ambulatory subspecialty clinics.

**Table 1:** Characteristics of focus group participants

Participant Characteristics	*N* (%)
Sex	
Female	9 (39.1%)
Male	14 (60.9%)
Title	
Resident Physician	
PGY-2	3 (13.0%)
PGY-3	3 (13.0%)
Instructor	3 (13.0%)
Assistant Professor	7 (30.4%)
Associate Professor	3 (13.0%)
Professor	2 (8.7%)
Non-Academic Appointment	2 (8.7%)
Specialties (excluding resident physicians)	
Primary Care	3 (13.0%)
Hospitalist Medicine	7 (30.4%)
Cardiology	2 (8.7%)
Endocrinology	1 (4.3%)
Gastroenterology	2 (8.7%)
Rheumatology	1 (4.3%)
Primary Teaching Environment (excluding resident physicians)	
Inpatient Cardiology and Medicine, BJH	5 (21.7%)
Inpatient Medicine, VA	7 (30.4%)
Outpatient Primary Care Rotation	5 (21.7%)

### Themes

**Fig. 1:**
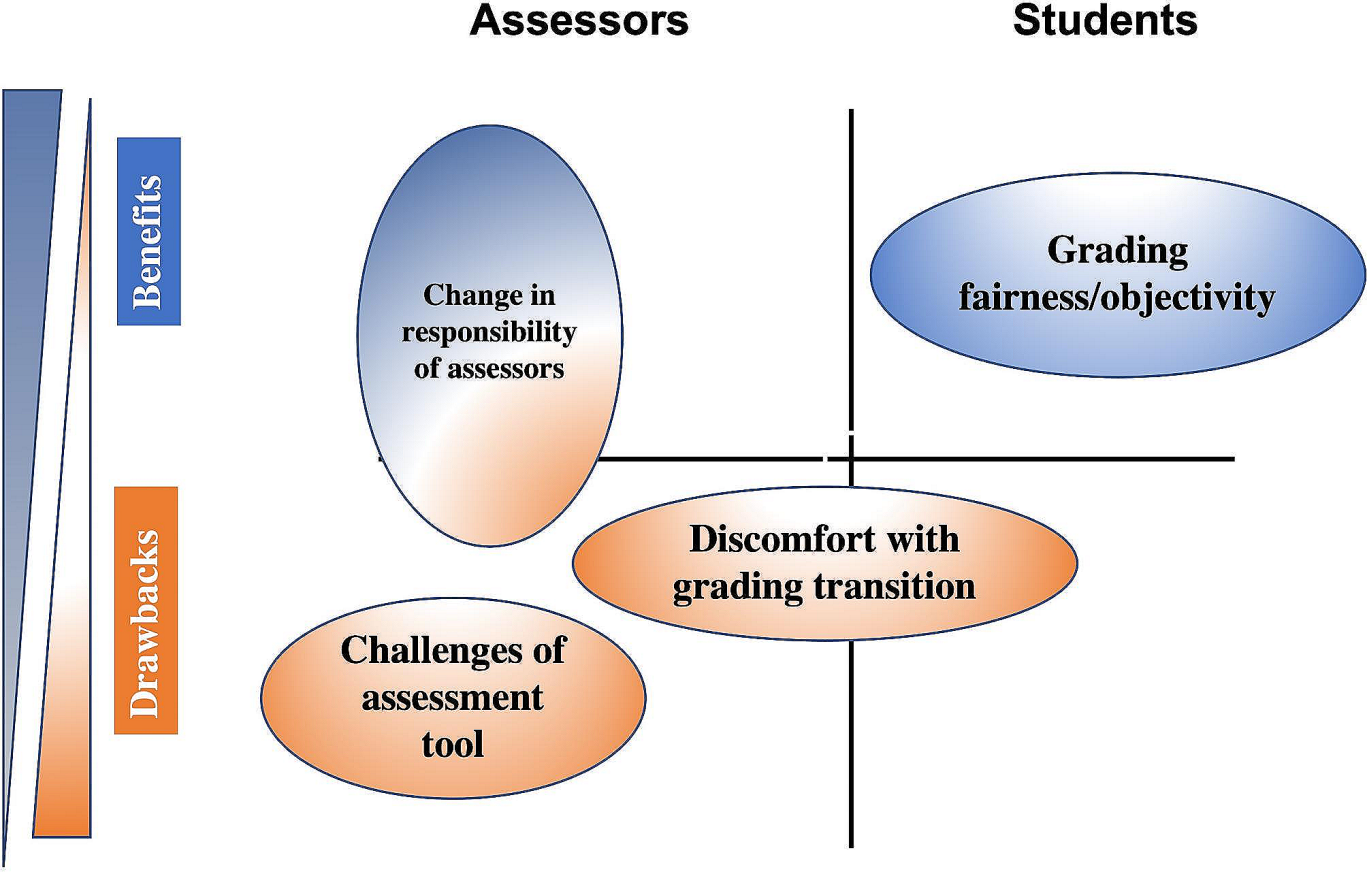
Summary of themes

Using thematic analysis, four themes emerged – grading fairness, change in responsibility of assessors, challenges of assessment tools, and discomfort with the grading committee transition (Fig. 1). Assessors view the switch from individual graders to a grading committee as theoretically beneficial to students due to increased grading fairness and beneficial to faculty due to decreased pressure. Despite these benefits, assessors report ongoing challenges in utilization of assessment tools and discomfort with the grading transition due to an incomplete understanding of the process.

#### Grading fairness

Participants universally agreed that switching from individual graders to a grading committee is beneficial, leading to a potentially fairer grading process. They cited that committee-assigned grading is “more standardized” and “objective” due to perceived decreased variability among graders and decreased impact of grader bias (Table 2, quote a). Some participants expressed concern that they may not have “enough exposure” and face time with students, especially on outpatient rotations where schedules may limit time observing and teaching students. They felt relieved that the grading committee takes into account perspectives from multiple assessors to provide a more complete picture of student performance (Table 2, quote a). Participants considered that the grading committee values how students’ skills “are growing over the course of” the clerkship, also contributing to a more comprehensive picture of student performance. Only one participant specifically cited that the grading committee evaluates students “blindly” after de-identifying assessment data, while most participants did not cite this factor.

**Table 2:** Coding and illustrative quotes: Grading Fairness

Subtheme	Codes	Illustrative Quotes
***Increased objectivity and standardization*** Grading committee decreases variability between assessors, standardizes process, reduces impact of unconscious bias	Benefits of grading committee, grader bias, grade inflation, assessor anxiety, limitations of Likert scales	**a.** “[In] the old system, there were people without much grading experience … individually grading each student. Now it seems that there are more trained, more specialized Grading Committees that then collect all of the data and use that [to] distribute grades in a more standardized fashion. Just by virtue of that alone, seems like the new system would be more beneficial and more objective than the old one.” (Resident)
***Grade Inflation*** Has benefit of possibly impacting culture of grade inflation but does not completely remove the tendency to inflate grades		**b.** “I’m actually happy not being involved in the grading part of it, and glad that it is now done anonymously… there’s so many circumstances in which a student is on the fence, and between pass and high pass, or between high pass, and honors…. I think that there’s always, at least for me, tendency to give the students the benefit of the doubt. I think that probably most people grade that way, and that inevitably leads to the grade inflation….” (Faculty) **c.** “I still feel pressure occasionally, when I get a learner who is … very vocal about finding out from me ahead of time what I think… I can still feel pressured from a learner to evaluate them in a certain way” (Faculty)

A subtheme that emerged from discussions of grading objectivity was grade inflation. Multiple interviewees discussed an institutional history of grade inflation, citing pressure from both students and the institution to provide favorable grades, as well as personal tendencies to “give students the benefit of the doubt” (Table 2, quote b, c). Participants expressed conflicting opinions of whether grading committees have the potential to relieve grade inflation. Several assessors noted that switching to a grading committee reduced pressure on faculty and residents to provide exaggeratedly positive assessments (Table 2, quote c) and mitigated the need to build strong, defensive arguments for issuing honest grades due to the more standardized grading process (Table 2, quote d). Other participants, however, cautioned that there could still be persistent pressure to provide overly positive feedback due to fear of being considered “overly mean” (Table 2, quote e).

#### Change in responsibility

The majority of participants reported a change of responsibility after WUSM transitioned to grading committees. Assessors commented that they “feel less involved with the grading aspect” because they are no longer recommending a grade, but instead “are more involved in…providing an assessment” because they are tasked with describing student behaviors relative to core objectives, while the grading committee interprets their descriptions to generate a grade. This was generally a welcomed change, resulting in more time to focus on student-centered feedback and less “pressure” put on clinical educators to give a final grade, a process that was almost universally considered to relieve stress (Table 3, quotes a-b). Several participants felt this new domain of responsibility for clinical educators was more in line with an ideal role of teaching faculty (Table 3, quote a).

**Table 3:** Coding and illustrative quotes: Change in responsibility of assessors

Subtheme	Codes	Illustrative Quotes
***Perceived positive change*** Less pressure and unwanted responsibility	Assessor anxiety, benefits of committee grading, value of assessment information drawbacks of committee grading, value of grading	**a.** “I definitely feel more comfortable in the new system feeling more that I can focus on being formative for them and giving them better feedback… Trying to be more […] like an advocate for their grade, as opposed to being the big, bad grader.” (Resident) **b.** “You don’t have to deal with any complaints […] when they don’t like their grade” (Faculty)
***Perceived negative change*** Some assessors want more of a voice in final grade		**c.** “There is something to be said about saying a grade and saying, if you were to distill everything down into one thing, where would you put this person? …. there’s something lost by not asking the person who was working with them at the bedside, where they thought they’d go.” (Faculty)

On the other hand, some participants felt that something was “lost” from grades no longer being assigned by the supervising resident and attending who spend the most face-to-face time with students, especially when it comes to students who are performing at the ends of the spectrum (Table 3, quote c). They wanted a chance to provide input on the final grade especially for “the students [who] should clearly get one grade or another,” such as for the outstanding or struggling students, but agreed that it is “nice to not necessarily have that responsibility” of assigning final grades for students whose performance may be borderline or unclear. Hospitalists, who most frequently assess learners on clerkship rotations, were most likely to identify a loss of voice in the final grading process.

#### Challenges of assessment tools

With the transition to grading committees, participants universally felt increased responsibility to provide detailed information of students’ performance, but they frequently cited barriers to providing high quality data via the 2020–2021 assessment forms. Interviewees generally agreed that faculty and resident time limitations were a major barrier to providing superior feedback (Table 4, quote a); however, participants harbored differing opinions on the relative technical challenges of the WUSM assessment tool, which incorporates both checklist responses and narrative feedback.

**Table 4:** Coding and illustrative quotes: Challenges of assessment tool

Subtheme	Codes	Illustrative Quotes
***Time constraints*** Leads to assessor fatigue and lack of thoughtful feedback	limitations of likert scales, limitations of narrative assessment, drawbacks of committee grading, assessor anxiety	**a.** “I just find the form really overwhelming” (Faculty)
***Limitations of checklist responses*** Difficulty communicating nuance, subtle differences between students and improvement over time		**b.** “I can definitely see how a lot of students would get the same evaluation […] You [could] end up with the same check boxes clicked, when maybe those students are in two different places […] The subtlety is lost” (Resident) **c.** “[Students can] have the same check boxes as everybody else. But when you actually talk to people who have worked with them, you get a very different picture. Check boxes alone […] are not adequate” (Faculty)
***Limitations of narrative feedback*** Unclear expectations and cumbersome		**d.** “It’s unclear to me, what is actually being asked, what kind of information is actually being sought in those [narrative] boxes.” (Faculty) **e.** “I don’t mind being asked what their strengths and what their weaknesses are, but […], there’s too many of those.” (Faculty) **f.** “You’re trying to figure out which combination of clicking these boxes is going to communicate this idea that I’m trying to communicate, when it would be easier to do a narrative, which is hard too, because a lot of people don’t like filling out narratives either on these evaluation forms.” (Faculty)

For some, the checklist responses addressing student performance across key domains suffered from a lack of “nuance.” Participants worried that outstanding students whose clinical performance exceeds expectations could appear the same on paper as students with consistent but average performance (Table 4, quote b). Conversely, many assessors struggled with communicating their assessment of students who simultaneously fulfilled performance checkboxes but still fell short of expectations for commendable performance (Table 4, quote c). They felt that an overall “gestalt” of a student was difficult to communicate using check boxes. For others, narrative assessments were overwhelming and repetitive, leading to assessment fatigue (Table 4, quote d-e). Participants recognized that the evaluation forms had multiple options to address these preferences (i.e. free text boxes to add nuance/context) but these were not uniformly acceptable or were too cumbersome for users (Table 4, quote f). Instead, some assessors indicated that they “would rather just talk to a human being,” such as the Clerkship Director, to provide narrative assessment in place of writing. Overall, participants believed they would benefit from training to improve the quality of their assessments to optimize the accurate communication of student performance to grading committees.

#### Discomfort with grading committee transition (and the need for training)

The use of grading committees created new sources of discomfort for assessors and concern it would lead to new sources of anxiety for students. Many participants noted apprehension regarding unfamiliarity with the new grading committee process (Table 5, quote a). They pointed out several areas of uncertainty including how committees utilize assessment forms to synthesize final grades, how one assessor’s evaluations are weighed relative to another, and the relative contribution of standardized exams and performance evaluations. There was a perceived lack of transparency and clarity in the grading committee process (Table 5, quote c). As one interviewee stated, they felt “in the dark” about how grading committee uses feedback. Several participants also “[perceived] some…increased anxiety” among medical students with the introduction of the grading committee. Students may perceive the grading process as “impersonal” without transparency and be apprehensive about what data is synthesized into a final grade, with what degree of importance.

**Table 5:** Coding and illustrative quotes: Discomfort with grading transition

Subtheme	Codes	Illustrative Quotes
***Assessor discomfort*** Lack of understanding of grading committee process, lack of formal training	Assessor anxiety, assessment training, understanding of assessment process	**a.** “I have to admit that I’ve sometimes gone into evaluations being unfamiliar with the process itself and it keeps changing […] I think that’s been part of our problem is we weren’t well instructed or well trained in what we were expected to do.” (Faculty) **b.** “There are sections on the form where I’m not sure what you’re going for. If we could have some sort of practical discussion with an example saying like, this is what this hypothetical student is doing clinically, and this is how we would like to see you fill out the evaluation form to reflect that, that would be helpful” (Faculty)
***Learner discomfort*** Students already have high anxiety around grading. Grading committee is impersonal.		**c.** “Whatever the committee does […] is behind the curtain” (Resident)

Participants generally had minimal or no prior formalized training in assessment and identified this as an additional area of discomfort (Table 5, quote a). While there was an assortment of topics that participants felt could be covered for faculty and resident development, they believed mandatory training on general topics such as assessment and feedback would be “less likely … to get a lot of buy-in from people (faculty and residents)” due to scheduling restraints and variable interest. Many participants, however, prioritized a need for “practical training” – specifically, increased guidance on how to complete high quality performance evaluations in order to communicate a comprehensive view of student performance to the receiving grading committee (Table 5, quote b). All focus groups agreed that training would ideally be delivered in a timely manner in close proximity to resident or faculty time on service. There was not a uniform opinion on the best format to disseminate training, but some frequent suggestions included a module describing how the committee interprets evaluation forms to come to a grading decision, a tutorial walking through the assessment form with a mock student, or an instructional video with “frequently asked questions” about the assessment form.

## Discussion

The fairness of medical student clerkship grades has been questioned due to the impact of bias, subjectivity, and interrater reliability. Grading committees and group decision-making are thought to promote grading consistency [6, 18, 19], especially when student data is reviewed in a de-identified manner. As evidenced by a recent survey of Clerkship Directors in Internal Medicine, many institutions are adopting grading committees as one strategy to improve grading equity [24]. Our study explores the opinions of faculty and resident assessors in the first year after transition to grading committees in the WUSM Internal Medicine clerkship. As we move toward grading committees, understanding assessors’ opinions about the process can facilitate implementation at other institutions, helping medical education leaders identify key stakeholders, lean into points of agreement, and prepare for points of dissent.

In this study, assessors unanimously agreed that group decision-making should improve standardization and help minimize the impact of bias and inter-assessor variability. Grading committees, however, are only one component to addressing issues of bias. Assessors can still write biased narratives, feel pressured to inflate evaluations, or demonstrate variable commitment to the submission of descriptive evaluations. Furthermore, grading committee members are still subject to inequities in the integration and prioritization of assessment data. This highlights the ongoing importance of implicit bias training for assessors and grading committee members, a practice that has not yet been universally adopted among medical schools [24].

For high quality assessments, there has also been a shift from personal commentary to behavior-based assessments in the form of clinical competencies, which are often assessed on a scale; however, rating scales are generally perceived to be poor motivators for student learning [25]. As a result, narrative comments remain a critical element of student evaluations, both to facilitate student development as well as to provide holistic context for performance. Narrative feedback can be, however, flawed and prone to stereotyped language [26]. Participants in our study highlighted the challenges of using assessment tools, identifying difficulty with accurate descriptions of performance via both narrative and multiple selection items. Some participants struggled to provide meaningful narrative feedback while others struggled to interpret the clinical competencies addressed on the ratings scales. A key take-away from our study is the importance of providing a diversity of mechanisms for assessors to share their observations, allowing assessors to utilize their strengths and preferences to provide the most accurate assessment data possible.

Participants wanted to raise the quality of assessment data they delivered to the grading committee. They believed that practical faculty/resident development sessions specifically geared at assessment could help achieve that goal, especially since most of our participants had no pre-existing formalized training in assessment methods. Notably, these requests for faculty development followed a clerkship-led effort to introduce assessors to the new grading committee role and how assessment forms would be utilized by the committee. These findings underscore the complexity of assessment strategies and reinforce the need for multi-modal, repeated faculty development initiatives at our institution and others.

When WUSM transitioned to the Gateway Curriculum in 2022, lessons learned from our study were incorporated into adapted assessment practices within the new curriculum. First, based on the feedback from this initiative as well as the focus on competency-based education, Gateway assessment forms have been streamlined, now comprised of 2–4 Likert scale questions and two boxes for narrative comments. The Gateway Internal Medicine clerkship addressed the challenge of narrative assessments by inviting assessors from inpatient rotations to a teleconference where clerkship leadership guide assessors through semi-structured interviews to provide assessment commentary. In exchange, these assessors are not required to submit written narrative comments. Second, in response to the viewpoints elucidated by this analysis, the Internal Medicine clerkship ramped up the development of frontline assessors’ assessment skills. It uses a multi-faceted approach to assessor development, incorporating didactic sessions, office hours, tip sheets, online modules, and personalized feedback. This approach provides options for assessors to learn the skills needed to assess students in the form they most prefer, and it is delivered iteratively throughout the year.

The strengths of our investigation reside within our methods. First, we encouraged honest responses from participants because peers, instead of members of clerkship leadership, conducted the semi-structured interviews. Second, we recruited a diversity of participants from residency, faculty, inpatient, and outpatient specialties. Lastly, our research team reinforced this diversity of perspectives, incorporating the perspectives of medical educators from residency training, faculty, and clerkship leadership into data analysis.

Our investigation has limitations. Our focus group participation rate was approximately 10% of total frontline assessors, although our estimate likely overapproximates the total number of individual assessors, thereby underestimating our participation rate. While we recruited a diversity of study participants, this relatively low participation rate may limit the generalizability of our results. Additionally, our focus group participants may represent a subset of assessors who have increased interest in medical education compared to the general population of assessors at a single institution, WUSM. We did not investigate if students perceive increased fairness after transitioning to grading committees nor did we include the perspective of grading committee members with respect to the quality or content of assessments. Therefore, we present a single viewpoint regarding the benefits and shortcomings of grading committees.

This study demonstrates that grading committees change the roles and responsibilities of frontline assessors, relieving the grading burden but increasing the emphasis on high quality written assessment, which is a persistent challenge. Faculty and resident development sessions focused on student assessment and constructive narrative feedback may better prepare our assessors for their roles. To this end, there is evidence that rater training can improve faculty confidence in clinical evaluation, however the impact on grading reliability is less clear [27–29]. More work needs to be done to determine if faculty development improves assessment quality or accuracy. Future investigation of grading outcomes after implementation of grading committees at WUSM is also needed to determine if this change enhanced equity.

### Electronic supplementary material

Below is the link to the electronic supplementary material.

Supplementary Material 1: Additional File 1. Interviewer Guide for Focus Group. File contains the interviewers’ guide for focus groups, including consent process and interview questions

## Data Availability

The datasets used and/or analyzed during the current study are available from the corresponding author on reasonable request.

## Abbreviations

BJH: Barnes-Jewish Hospital
VAMC: John Cochrane Veterans Affairs Medical Center
WUSM: Washington University School of Medicine in St. Louis
