# Supplementary material for: Frontline assessors’ opinions about grading committees in a medicine clerkship
Source: BMC Med Educ. 2024 Jun 5;24:620. doi: 10.1186/s12909-024-05604-x (PMC11151467; doi:10.1186/s12909-024-05604-x)
Supplement: Supplementary file 1 — Supplementary Material 1: Additional File 1. Interviewer Guide for Focus Group. File contains the interviewers’ guide for focus groups, including consent process and interview questions [file 12909_2024_5604_MOESM1_ESM.pdf]

Additional File 1: Interviewer Guide for Focus Group

What are frontline assessors' opinions about the medicine clerkship's assessment and grading process?

For the Interviewer: The focus group will cover the topics listed in this interview guide in an organic way. Questions may not be phrased exactly as written or asked in the order presented in this guide. Instead, the guide represents the key topics that will be covered in the focus group and examples of the questions that might be asked. In the event that participants raise unanticipated topics, the researchers will decide whether they are relevant to the study and if they should be explored with further questioning.

Introduction to the Focus Group

> Thank you for participating in today's focus group and for sharing your opinions on frontline assessors' role in assessing and grading medical students. The opinions you share today will help us improve our approach to the clerkship's assessment and grading process.
>
> We will record today's zoom session so that we can transcribe your thoughts for future reference, but we will remove identifying information from the transcript to ensure that the opinions that you share today will remain anonymous and confidential. After the recording is transcribed, it will be deleted. We also may reach out to you for further clarification on points discussed during this session as we analyze the transcript. Is everyone in agreement with this plan?
>
> Let's briefly introduce ourselves to each other. Please share your name, current job title, and involvement in the medicine clerkship.

	**Leading Question**	**Possible Follow-up/Probing Questions**
1)	How do you define "assessment" and "grading?"	What do these terms mean to you?<br>How are they similar?<br>How are they different?<br>How would you explain to a student what the difference is between "assessment" and "grading?"
2)	What is your understanding of the current medicine clerkship assessment and grading process and your role in that process?	*Provide an explanation if the group does not understand the new assessment and grading process (i.e. frontline assessors → feedback → grading committee)*
3)	What role should faculty and residents play in assessing and grading medicine clerkship students?	*Make sure the group comments on both faculty and residents before focusing on one group (i.e. faculty or residents).*
4)	How does your current role in the assessment and grading of medicine clerkship students differ from what you believe your role should be?	Are you satisfied with the clerkship's transition to a grading committee? Why?<br>Are you dissatisfied with the clerkship's transition to a grading committee? Why?
5)	What are the benefits of the clerkship's new assessment and grading process?	*Allow questions 5-6 to bring out the concepts of fairness, unconscious bias, balancing roles as assessor and educator, etc. as they relate to students and/or assessors.*
6)	What are the challenges or shortcomings of the clerkship's new assessment and grading process?	
7)	In your opinion, what is the students' understanding of the current assessment and grading process?	Have you heard the students comment on the current process?<br>Have you observed a change in medical students' anxiety level, behavior, or approach to the current process?
8)	How would you explain the new assessment and grading process to students rotating through the clerkship?	Why would you explain it in this way?<br>What mechanisms would you use to provide the explanation? Orientation? Email? etc

9) Have you had previous training in clinical assessment and grading?	*Optional question if time allows*<br>Tell me about that training. When was it? Who provided it? How long ago? Delivery method?
10) Describe the ideal training session for faculty and residents to learn how to assess medicine clerkship students.	What topics would you include?<br>What aspects of the current assessment and grading methods need to be emphasized?<br>How much time would you devote to the session? Delivery method?<br>Frequency of repeated training?
